# New Strategies to Prolong the In Vivo Life Span of Iron-Based Contrast Agents for MRI

**DOI:** 10.1371/journal.pone.0078542

**Published:** 2013-10-25

**Authors:** Antonella Antonelli, Carla Sfara, Serafina Battistelli, Barbara Canonico, Marcella Arcangeletti, Elisabetta Manuali, Sonia Salamida, Stefano Papa, Mauro Magnani

**Affiliations:** 1 Department of Biomolecular Sciences, Biochemistry and Molecular Biology Section, University of Urbino “Carlo Bo”, Urbino, Italy; 2 Department of Biomolecular Sciences, Clinical Biochemistry Section, University of Urbino “Carlo Bo”, Urbino, Italy; 3 Department of Earth, Life and Environmental Sciences, University of Urbino “Carlo Bo”, Loc. Crocicchia, Urbino, Italy; 4 Department of Biomolecular Sciences, Pharmacology and Pharmacognosy Section, University of Urbino “Carlo Bo”, Urbino, Italy; 5 Laboratory of Electron Microscopy & Histopathology, Experimental Zooprofilactic Institute of Umbria & Marche, Perugia, Italy; University of California Davis, United States of America

## Abstract

Superparamagnetic iron oxide (SPIO) and ultra small superparamagnetic iron oxide (USPIO) nanoparticles have been developed as magnetic resonance imaging (MRI) contrast agents. Iron oxide nanoparticles, that become superparamagnetic if the core particle diameter is ^~^ 30nm or less, present R1 and R2 relaxivities which are much higher than those of conventional paramagnetic gadolinium chelates. Generally, these magnetic particles are coated with biocompatible polymers that prevent the agglomeration of the colloidal suspension and improve their blood distribution profile. In spite of their potential as MRI blood contrast agents, the biomedical application of iron oxide nanoparticles is still limited because of their intravascular half-life of only few hours; such nanoparticles are rapidly cleared from the bloodstream by macrophages of the reticulo-endothelial system (RES). To increase the life span of these MRI contrast agents in the bloodstream we proposed the encapsulation of SPIO nanoparticles in red blood cells (RBCs) through the transient opening of cell membrane pores. We have recently reported results obtained by applying our loading procedure to several SPIO nanoparticles with different chemical physical characteristics such as size and coating agent. In the current investigation we showed that the life span of iron-based contrast agents in the mice bloodstream was prolonged to 12 days after the intravenous injection of murine SPIO-loaded RBCs. Furthermore, we developed an animal model that implicates the pretreatment of animals with clodronate to induce a transient suppression of tissue macrophages, followed by the injection of human SPIO-loaded RBCs which make it possible to encapsulate nanoparticle concentrations (5.3-16.7mM Fe) higher than murine SPIO-loaded RBCs (1.4-3.55mM Fe). The data showed that, when human RBCs are used as more capable SPIO nanoparticle containers combined with a depletion of tissue macrophages, Fe concentration in animal blood is 2-3 times higher than iron concentration obtained by the use of murine SPIO-loaded RBCs.

## Introduction

Superparamagnetic iron oxide (SPIO) particles are iron oxide nanocrystals with different core materials, magnetite (Fe_3_O_4_) or maghemite (γ-Fe_2_O_3_), different hydrodynamic diameters and different coatings such as polyethylene glycol (PEG), dextran, chitosan, silica, phospholipids, generally used as biocompatible materials to improve in vivo stability [1-3]. 

For the past two decades, dextran coated SPIO particles such as AMI-227 (Sinerem^®^), AMI-25 (Endorem^®^) or carboxydextran coated particles such as SHU 555A (Resovist^®^) have served as contrast-enhancing probes for MRI, although today they are no longer commercially available [4,5]. Magnetic iron oxide particles appear to yield higher detection sensitivities than gadolinium complexes; however, in molecular imaging they have the disadvantage of being rapidly internalized by phagocytosis. In fact, following intravenous administration, SPIOs are rapidly coated by serum proteins [6,7]. This opsonization process renders the particles recognizable by the body’s major defence system, the RES. Since the macrophage cells of the liver, spleen and lymph tissues play a critical role in the removal of opsonized particles, the SPIO nanoparticles are commonly used for MRI analysis of these organs [8]. In literature there are reports of both coating nanoparticles with different molecules and strategies that attempt to inhibit the opsonization by plasma components, thereby permitting longer circulation times [9-12]. Although surface modifications and size reductions have been explored to prolong the blood half-life of these contrast agents, their application in MRI, especially MRI of the circulatory system, is often compromised by RES uptake. Indeed, the resulting time frame for nanoparticle bolus-based measurements is very short, only a few minutes [13,14]. Advances in nanotechnology and molecular cell biology have led to improved stability and biocompatibility of iron oxide-based nanoparticles, but SPIO survival in the bloodstream is still limited. Herein we propose the use of red blood cells (RBCs) as carriers of SPIO nanoparticles to obtain a blood pool tracer with longer blood retention time [15]. RBCs, thanks to their unique properties which allow them to be reversibly opened under hypotonic conditions without losing their natural features and functionalities, can be used as intravascular carriers for different bioactive substances including drugs, therapeutic proteins, nucleotide analogues, cancer chemotherapeutics and nucleic acids [16].

We have previously demonstrated that it is possible to encapsulate different SPIO nanoparticles in human and murine RBCs. These nanoparticles are monodispersed and sufficiently small to cross the RBC membrane pores opened by the reduction of the physiological osmolarity. In this context, SPIO-loaded RBCs offer the advantage of being able to survive for a number of days without being eliminated, with a life span which is comparable to that of native cells [15]. Nevertheless, we have previously shown that not all iron oxide nanoparticles can be loaded into RBCs. In fact, encapsulation depends on several factors such as the type of dispersant agent and the nature and size of the nanoparticles [17].

Recent studies have investigated the potential of RBCs loaded with SPIO nanoparticles as a tracer material for magnetic particle imaging (MPI), a novel medical imaging technique introduced by Philips. These studies provided evidence of the feasibility of detecting a heart beat in mice after intravenous injection of murine Resovist^®^-loaded RBCs [18,19]. Indeed, 3h after injection, a clear signature of the beating heart, and thus of the signal generated from loaded RBCs still flowing in the blood, can be detected. Moreover, 24h after the injection, the iron concentration in the blood is close to the detection limit of the scanner prototype [20].

In order to increase SPIO concentrations in animal blood circulation we have developed a new procedure that involves the infusion of human SPIO-loaded RBCs chosen for their ability to contain higher nanoparticle concentrations. Before the infusion, the animals were treated with clodronate, a potent bisphosphonate (BP) generally used to inhibit osteoclastic bone resorption which is able to induce a reversible depletion of tissue macrophages. BPs have also been shown to affect the survival of macrophages that are ontogenetically related to osteoclasts and under certain conditions share functional homology with these primary bone-resorbing cells [21]. The clodronate is metabolized by target cells to AppCCl_2_p (adenosine 5’-[β,γ-dichloromethylene] triphosphate), a nonhydrolyzable analog of ATP which has a cytotoxic effect on mature osteoclasts inducing cell death by apoptosis. The accumulation of the ATP analog is likely to inhibit numerous ATP-dependent intracellular metabolic enzymes, thus having detrimental effects on cell metabolism and survival [22,23]. In this context, a pretreatment of animals with clodronate leads to a temporary blockade of phagocytosis by Kupffer cells, and hence might prevent the rapid clearance of human SPIO-loaded RBC constructs improving their survival in the bloodstream circulation.

The presence of murine or human SPIO-loaded RBCs injected into animals which were pretreated with clodronate or not treated with the drug was investigated by Nuclear Magnetic Resonance (NMR) and/or cytofluorimetric analysis. 

The use of longer lasting blood half-time tracer materials such as SPIO-loaded RBCs may provide an opportunity to improve interventional procedures and long-term monitoring of cardiovascular diseases in applications that require continuous imaging of the vessel tree or other blood filled structures since up to now the time imaging window for data acquisition is limited to the first pass due to the very short life span of iron oxide nanoparticles. The prolonging of SPIO nanoparticle life span using stable RBC carriers could significantly aid MRI and MPI diagnostics since it would allow the patient to be imaged on number of occasions over time.

## Materials and Methods

### 2.1. Materials

The RBC loading procedure was carried out with the following suspensions of SPIO contrast agents:

I. Resovist^®^ or SHU 555A (540 mg/ml of ferucarbotran, corresponding to 28 mg Fe/ml or 0.5 mmol/ml) by Bayer Schering Pharma, consists of an aqueous suspension of superparamagnetic iron oxide nanoparticles that are carboxydextran-coated with a mean hydrodynamic diameter of 62 nm and core diameter of 4.2 nm [24].
II. Endorem^®^ or AMI-25 (Feridex^®^, 11 mg Fe/ml or 0.2 mmol/ml) by Guerbet (Roissy, France), consisting of iron oxide nanoparticles dextran-coated with a hydrodynamic diameter of 80-150 nm and core of 4.8-5.6 nm [25].
III. Sinerem^®^ or AMI-227 (Combidex^®^, 20 mg Fe/ml or 0.357 mmol/ml) by Guerbet (Roissy, France) composed of a crystalline iron oxide core of 4-6 nm surrounded by dextran coating. The hydrodynamic diameter is 20-40 nm [26].

Clodronate (Cl_2_MDP, dichloromethylene bisphosphonate disodium salt) was purchased from SIGMA Aldrich (Italy). A stock solution of clodronate was prepared by dissolving clodronate in phosphate buffer saline (PBS) at a concentration of 25mg/ml or 86.5 mM. 6.0M sodium hydroxide was used to adjust the pH 7.4 and the solution was filter-sterilized (0.2-µm syringe filter, Nalgene, Rochester, NY) prior to use.

### 2.2. In vitro studies

#### 2.2.1. Loading procedure to encapsulate SPIO nanoparticles in RBCs

Human blood was obtained from the Blood Transfusion Center of the Hospital “S. Maria della Misericordia” - Urbino, Italy. Blood was provided by healthy adult volunteers who signed an informed consent form before donation and samples were collected anonymously in heparinised tubes. The use of human blood in the present study was approved by the research ethics committee of the University of Urbino.

RBCs from freshly drawn blood were isolated by centrifugation at 1400g at 4°C for 10 min. The serum and buffy coat were removed and the packed cells were washed three times with Hepes buffer (10 mM Hepes, 154 mM NaCl, 5 mM glucose, pH 7.4) and then resuspended at 70% haematocrit in the same buffer. 1ml of these cells were dialysed in the presence of Resovist^®^, Sinerem^®^ or Endorem^®^ contrast agents ranging from 5.6 mg to 22.4 mg Fe, for 75 min using a tube with a 12-14 kDa cut-off in 50 vol of a dialysis buffer (10 mM NaHCO_3_, 10 mM NaH_2_PO_4_, 20 mM glucose, 4 mM MgCl_2_ pH 7.4), containing 2 mM ATP and 3 mM reduced glutathione. The osmolarity of the dialysis buffer was 64mOsm. All these procedures were performed at 4°C under sterile conditions. Resealing of the RBCs was obtained by adding 0.1 vol of PIGPA (5 mM adenine, 100 mM inosine, 2 mM ATP, 100 mM glucose, 100 mM sodium pyruvate, 4 mM MgCl_2_, 194 mM NaCl, 1.606 M KCl, 35 mM NaH_2_PO_4_, pH 7.4) per vol of dialysed RBCs and by incubating at 37°C for 45 min. The resealed cells were recovered by centrifugation at 400g and washed four times with Hepes buffer to remove unentrapped magnetic particles. Unloaded RBCs were prepared following the same procedure, but were dialysed in the absence of magnetic material.

The encapsulation of magnetic nanoparticles in murine RBCs was performed using the same procedure with a few modifications: 1ml of murine RBCs at 70% haematocrit was dialysed in the presence of Resovist^®^, Sinerem^®^, or Endorem^®^ contrast agents ranging from 2.8 mg to 5.6 mg Fe. The murine blood withdrawn from ocular arteria was centrifuged at 400 g and the osmolarity of the dialysis buffer (15 mM NaHCO_3_, 15 mM NaH_2_PO_4_, 20 mM glucose, 4 mM MgCl_2_ pH 7.4) was 88 mOsm. 

#### 2.2.2. Cell integrity

To determine whether the loaded cells retained the properties of native RBCs, several features of cell integrity were examined. Mean corpuscolar volume (MCV), mean hemoglobin concentration (MCH) and mean corpuscolar hemoglobin concentration (MCHC) were measured with an automated hemocytometer (Model MICROS O.T, Horiba ABX Diagnostics, Italy). The hemocytometer was also used to determine the number of total intact RBCs before and after magnetic nanoparticle loading to determine the percentage of cell recovery.

#### 2.2.3. Morphology

Normal, unloaded and loaded RBCs were examined by Transmission Electron Microscopy (TEM). Cells were quickly washed in 0.1 M Sörensen phosphate buffer pH 7.3, sedimented at 600 g and immediately fixed in 2.5% glutaraldehyde in the same buffer for 1h.

The cells were then postfixed in 1% OsO_4_ in phosphate buffer, dehydrated with ethanol and embedded in Epon 812. Ultrathin sections were collected on nickel grids, stained with uranyl acetate and lead citrate, and analyzed with a Philips CM10 electron microscope operating at 80 kV.

#### 2.2.4. NMR relaxation measurements

The NMR longitudinal (T1) and transverse (T2) relaxation times of all SPIO-loaded RBC samples at 44% of hematocrit were measured at a magnetic field strength of 4.7 tesla at 37°C using an AC-200 NMR-Bruker spectrometer. T1 was determined using a 180°-τ-90° inversion recovery sequence with a fixed relaxation delay of at least 5 x T1 [27]. The times of inversion (τ) were chosen on the basis of an estimated T1 value. T2 was measured using the Carr-Purcell-Meiboom-Gill (CPMG) method [28,29]. The echo-times were chosen on the basis of an estimated T2 value.

The magnetite concentration in SPIO loaded RBCs was determined by NMR relaxation measurements using a dose-response curve generated by adding known amounts of Resovist^®^, Endorem^®^ or Sinerem^®^ contrast agents to human or murine blood samples. The iron content values obtained by NMR measurements are only approximate and are used only for relative concentrations encapsulated in RBCs. 

The values of (1/T1_c_ - 1/T1_0_) (where T1_c_ is the relaxation time at the concentration [c] of contrast agent and T1_0_ the relaxation time of the RBCs sample without contrast agent) were plotted versus the concentration of contrast agent and were fitted by the least squares method to a straight line, the slope of which is the longitudinal relaxivity (r1). r1 of human RBCs added to Resovist^®^, Endorem^®^ or Sinerem^®^ contrast agents was calculated in the concentration interval 0mM<[c]<15mM Fe with at least 9 concentration levels. The transversal relaxivity (r2) was calculated in the concentration intervals 0mM<[c]<0.3mM Fe for Resovist^®^, 0mM<[c]<3mM Fe for Sinerem^®^ and 0mM<[c]<2.5mM Fe for Endorem^®^ with at least 6 concentration levels.

r1 of murine RBCs added to Resovist^®^ or Endorem^®^ contrast agents was calculated in the concentration interval 0 mM<[c]<10mM Fe with at least 8 concentration levels and for Sinerem^®^ in the range 0mM<[c]<15mM Fe with 9 concentration levels while r2 in the interval 0mM<[c]<5mM Fe for Endorem^®^ with at least 7 concentration levels, 0 mM<[c]<3 mM Fe for Resovist^®^ at least 6 concentration levels, and 0 mM<[c]<0.6 for Sinerem^®^ at least 4 concentration levels.

**Figure 1:**
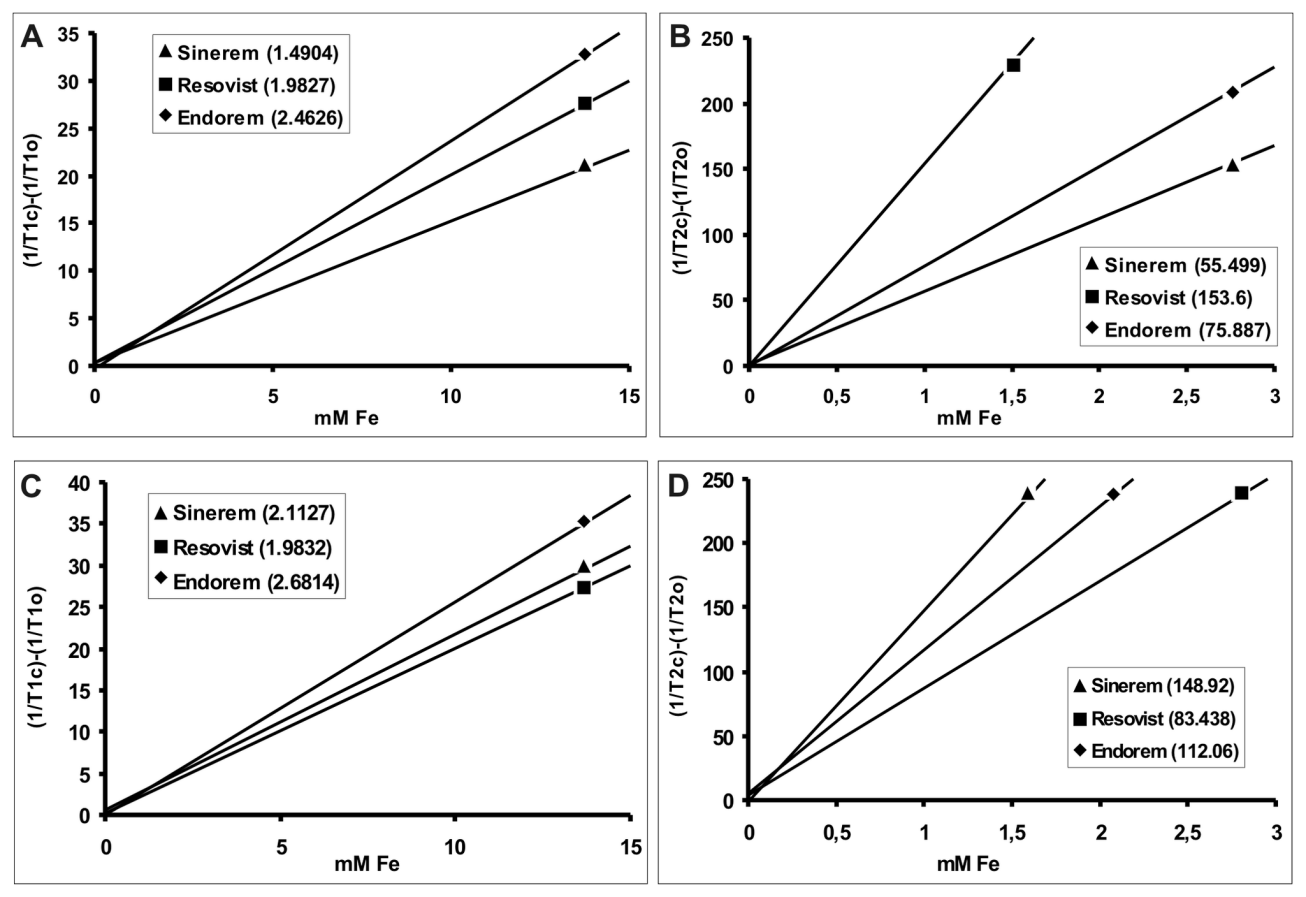
Relaxivity constants r1 or r2 of SPIO nanoparticles added to RBCs. r1 (**A**) and r2 (**B**) relaxivity constants of SPIO nanoparticles added to human RBCs. r1 (**C**) and r2 (**D**) relaxivity constants of SPIO nanoparticles added to murine RBCs. Exact r1 and r2 values are reported in parentheses next to the name of the material encapsulated in the RBCs.

Relaxivity constants (r1 and r2) of Resovist^®^, Endorem^®^ or Sinerem^®^ added to human or murine RBC samples at 44% of hematocrit are reported in Figure 1. r1 and r2 relaxivities represented by the lines, were obtained as least squares fit through the data points and the slopes. For the correlation to be considered linear, the following criteria were defined: correlation coefficient (R squared) greater than 0.990 and the distribution of points around the regression curve must be randomly distributed. Consequently, it was possible to estimate the concentrations of Resovist^®^, Endorem^®^ or Sinerem^®^ encapsulated in human and murine RBCs by using the inverse formula [c]=(1/T1_c_ - 1/T1_0_)/r1 and [c]=(1/T^2^_c_ - 1/T^2^_0_)/r2 in correspondence of the measured T1_c_ and T2_c_ values of loaded RBC suspensions at 44% of hematocrit.

### 2.3. In vivo studies

#### 2.3.1. Ethics statement

Housing and treatment of mice and rats were in compliance with the recommendations in the Guide for the Care and Use of Laboratory Animals by the Health Ministry, law 116, 1992. Experiments were approved by the Committee on the Ethics of Animal Experiments of the University of Urbino ‘‘Carlo Bo’’ – Italy (Prot. CESA 3/2012, on September,14, 2012). 

#### 2.3.2. Animals

Female six-week-old ICR (CD-1^®^) mice (Harlan, Italy) weighing 32 g or Sprague Dawley^®^ SD^®^ rats (Harlan, Italy) weighing 415-430 g were used. The animals were housed at 22 ± 1°C with a 12-h light/dark cycle, 60 ± 5% humidity, and 12 air changes/h. Food and water were given ad libitum. To minimize pain and stress, the animals were anesthetized for the blood draw and the intraperitoneal (i.p.) or intravenous (i.v.) injections. Mice were anesthetized with CO_2_ and rats with 5% isoflurane in N_2_O/O_2_ (70/30%) mixture. 

#### 2.3.3. Administration of murine Resovist^®^-loaded RBCs in mice

First, *in vivo* experiments on mice were performed to evaluate the presence in the vascular system of murine Resovist^®^-loaded RBCs prepared as previously described. Briefly, 4.8 ml of RBCs at 70% of hematocrit were dialysed in the presence of 960 µl of Resovist^®^ corresponding to 5.6 mg Fe/ml RBCs.

Three mice received a single bolus injection in the tail vein of 500 μl of a murine Resovist^®^-loaded RBCs suspension (3mM Fe) at 44% hematocrit corresponding to 46.8 μmol Fe/kg (Table 1, Experiment I, Group III). Three other mice received a single bolus injection of bulk Resovist^®^ suspension at the same concentration (Table 1, Experiment I, Group II).

**Table 1:** Design of *in vivo* experiments.

Experiment *I* Pharmacokinetic of murine Resovist^®^-loaded RBCs in mice					
**Mice**	Treatment			Analyses of blood samples	
Group I	None			NMR	
Group II	bulk Resovist^®^				
Group III	Resovist^®^-loaded RBCs				
Experiment *II* Human Sinerem^®^-loaded RBCs: in vivo studies in mice					
**Mice**	Treatment		Analyses of blood samples		Analyses of tissues
Group I	None	NMR			AAS, Perl’s staining, F4/80 staining
Group II	Unloaded RBCs				
Group III	Clodronate pretreated + unloaded RBCs				
Group IV	Sinerem^®^-loaded RBCs				
Group V	Clodronate pretreated + Sinerem^®^-loaded RBCs				
Experiment *III* Human Sinerem^®^-loaded RBCs: in vivo studies in rats					
**Rats**	Treatment			Analyses of blood samples	
Group I	None			NMR, Flow cytometry	
Group II	Unloaded RBCs				
Group III	Sinerem^®^-loaded RBCs				
Group IV	Clodronate pretreated + Sinerem^®^-loaded RBCs				

Experiment *I* was designed to evaluate murine Resovist^®^-loaded RBC survival in the bloodstream of mice after their intravenous injection compared to the life span of bulk Resovist^®^ suspension. Experiment *II* and Experiment *III* consider the use of human Sinerem^®^-loaded RBCs as larger SPIO containers and the pretreatment of mice or rats with clodronate to obtain a temporary depletion of tissue macrophages in order to increase Fe concentrations in the animal bloodstream.

NMR measurements of T1 and T2 relaxation times, measured as reported in “NMR relaxation measurements”, were performed on blood samples drawn at 0, 1, 4, 24, 48, 120, 168, 216 and 288 hours post injection to monitor survival of murine Resovist^®^-loaded RBCs in the bloodstream compared to the survival time of bulk Resovist^®^.

#### 2.3.4. Administration of human SPIO-loaded RBCs in mice pretreated with clodronate or not treated with the drug

The treatment of mice with clodronate was performed by i.p. injections of 360 μl of a clodronate solution 3.46mM concentrated corresponding to 11 mg clodronate/kg mouse. Untreated mice and PBS treated mice were used as controls. Both clodronate and PBS solutions were administered three times at 3 day intervals up to 24h before the i.v. injection of human unloaded or Sinerem^®^-loaded RBCs at 44% hematocrit.

Human Sinerem^®^-loaded RBCs were prepared by dialysing 6.5 ml of RBCs70% in the presence of 5.8 ml Sinerem^®^, corresponding to 18 mg Fe/ml RBCs. 

Fifteen mice were randomly assigned to 5 groups. One control group of untreated mice (Group I, Experiment II, Table 1) and 4 different groups of treatment: mice i.p. pretreated with PBS before the i.v. injection of unloaded human RBCs (Group II, Experiment II, Table 1), mice i.p. pretreated with clodronate before the i.v. injection of unloaded human RBCs (Group III, Experiment II, Table 1), mice i.p. pretreated with PBS, receiving the i.v. injection of 800 μl of human Sinerem^®^-loaded RBCs (11.7 mM Fe), corresponding to 285 μmol Fe/kg (Group IV, Experiment II, Table 1), mice i.p. pretreated with clodronate and receiving the i.v. injection of 800 μl of human Sinerem^®^-loaded RBCs (11.7 mM Fe) (Group V, Experiment II, Table 1). There were three mice in each group.

At 24-hours post injection of human unloaded or Sinerem^®^-loaded RBCs, blood was collected from the mice to perform NMR relaxation times measurements. The mice were then euthanized and their organs (livers, spleens and kidneys) were processed for histological examinations and atomic absorption spectrometry (AAS) analysis.

#### 2.3.5. Administration of human SPIO-loaded RBCs in rats pretreated with clodronate or not treated with the drug

Clodronate administration was performed by i.p injecting 1ml of 86.5 mM clodronate solution corresponding to 62 mg clodronate /kg rat. The drug was administered three times at 3 day intervals for each rat up to 24h before the i.v. injection of human unloaded or Sinerem^®^-loaded RBCs at 44% hematocrit. 

Human Sinerem^®^-loaded RBCs were prepared dialysing 5.5 ml of human RBCs70% in the presence of 2.3 ml of Sinerem^®^, corresponding to 8.5 mg Fe/ml RBCs.

Twelve rats were randomly assigned to 4 groups. One control group of untreated rats (Group I, Experiment III, Table 1) and three different treatment groups: rats receiving i.v injection of 1.8 ml of human unloaded RBCs (Group II, Experiment III, Table 1), rats receiving i.v injection of 1.8 ml of human Sinerem^®^-loaded RBCs (5.7 mM) corresponding to 24.4 μmol Fe/kg (Group III, Experiment III, Table 1), rats i.p. pretreated with clodronate before receiving i.v. injection of 1.8 ml of human Sinerem^®^-loaded RBCs (5.7 mM) (Group IV, Experiment III, Table 1). There were three rats in each group. After treatment, blood samples were drawn at different times (24, 48, 120 and 168 hours) by making a small incision at the end of the tail of anesthetized rats, and the presence of human SPIO-loaded RBCs in the animal bloodstream was evaluated both by NMR measurements and flow cytometry analysis. 

#### 2.3.6. Histology

Liver and spleen specimens of mice were collected and fixed in 10% neutral buffered formalin, dehydrated in a graded series of ethanol, processed and embedded in paraffin wax.

4 μm thick sections were then cut with a microtome and stained with haematoxylin-eosin (H-E). Perls Prussian Blue method for iron staining was performed using 4 μm sections that were hydrated using xylene and a graded alcohol series. The sections were then incubated with 2% aqueous hydrochloric acid (Sigma-Aldrich) and 2% aqueous potassium ferrocyanide (Carlo Erba) for 15 min, washed for 5 min in distilled water and counterstained with filtered 1% aqueous neutral red stain (Sigma-Aldrich) for 3 min. After being washed with distilled water, the sections were rapidly dehydrated in absolute alcohol, cleared and mounted in a DPX mountant (Sigma-Aldrich). A known positive control section was included in each staining.

The evaluation of macrophage depletion obtained by clodronate treatment was carried out by immunohistochemistry studies, using a monoclonal antibody recognizing F4/80 antigen, a glycoprotein expressed by mature murine macrophages [30]. 

For this evaluation, 4 μm sections were cut with microtome and mounted on poly-L-lysine-coated glass slides and deparaffinised. Endogenous peroxidase (activity) was blocked using 1% hydrogen peroxide in PBS with 10% methanol for 15 min.

Citrate buffer 10mM at pH 0.6 in microwave (2 x 5 min at 750 W) was used for antigen retrieval. 

After three washes in 0.1M PBS pH 7.4, slides were blocked with 10% goat normal serum in PBS for 15 min at room temperature and incubated overnight at 4°C with a rat anti-mouse F4/80 antigen (BioLegend, San Diego, USA) diluted 1:50 in PBS containing 1% BSA. 

Slides were then washed three times with PBS and biotinylated goat anti-rat IgG antibody (Vector Laboratories, Burlingame, USA) diluted 1:100 in PBS with 1% BSA added.

After further washing, the avidin-biotinylated horseradish peroxidase complex (ABC kit, Vectastain, Vector Laboratories, CA, USA) was used by incubation for 30 min followed by washing in PBS (3x 5 min). Diaminobenzidine (Sigma Chemical CO, St. Louis, USA) with 0.03% hydrogen peroxide was applied for 5 min. Counterstaining was performed with Mayer’s hematoxilin for 3 min. Negative control sections incubated without F4/80 antibody were included. 

To evaluate the number of macrophages, ten fields/slides were examined for each sample.

#### 2.3.7. Iron quantitative analysis

Livers and spleens were weighed, placed in appropriate teflon vessels and dried at 60°C for 48 h in a microwave oven, MDS-2100, CEM (CEM Corporation, Matthews, NC, USA). A mixture of nitric acid (Suprapur^®^ 65%, Merck, Darmstadt, Germany) and H_2_O_2_ (Suprapur^®^ 30%, Merck) was added to the dried tissues for tissue digestion. AAS readings of digested tissues were performed to quantify iron concentration (µg/g). An Aanalyst-300 atomic absorption spectrometer (PerkinElmer, Shelton, CT, USA) equipped with a deuterium arc lamp background correction system was used. Iron content was determined by flame atomic absorption spectrometry (FAAS). The accuracy of the sample preparation method was checked using Fe haemoglobin that was read in triplicate at known concentrations. The mean recovery percentages were 96.23 %.

#### 2.3.8. Flow cytometry

Flow cytometric determinations of human Sinerem^®^-loaded RBCs percentages circulating in the animal bloodstream, were performed immediately after injection of human Sinerem^®^-loaded RBCs prepared from human blood group A, monoclonal FITC Mouse-Anti-human blood group A (clone NaM87-1F6, BD Biosciences, San Jose, CA, USA) was used to mark human RBCs.

A FACS calibur flow cytometer, equipped with a 15 mW 488 nm, air-cooled argon-ion laser and a second red diode laser, 635 nm (Becton Dickinson, San Jose, CA, USA), with CellQuest^TM^ Software was used. Preliminary *in vitro* tests to define optimal concentration of antibody and to assess its affinity to group A human RBCs were carried out. The human untreated or SPIO-loaded RBCs were found to be 99% positive for group A-antigen, while rat and mouse RBCs were negative for the anti-human blood group A. Following intravenous injection of human Sinerem^®^-loaded RBCs, blood samples from mice or rats were taken at different times; 0.5 μg of anti A-mAbs were added to 1 x 10^6^ RBCs in 50 μl Hepes, gently mixed and incubated 15 min at room temperature in the dark before performing the flow cytometric analyses. 

#### 2.3.9. Statistical analysis

Statistical analysis of data was performed by ANOVA for repeated measurement, followed by Bonferroni multiple comparison test (for n groups >2) or with the Student t-test (for n groups = 2), using the GraphPad InStat version 3.0.6 for Windows (GraphPad Software). Differences between values were considered statistically significant at p ≤ 0.05 (*), very significant at p<0.01 (**) and extremely significant at p<0.001 (***).

## Results

### 3.1. Encapsulation of magnetic nanoparticles in human and murine RBCs

The total preparation procedure resulted in a cell recovery of human Resovist^®^, Endorem^®^ or Sinerem^®^-loaded RBCs ranging from 60 to 70%, which was similar to the recovery rate for unloaded cells. Several parameters of RBCs, such as MCV, MCH, MCHC, were measured in order to evaluate the cell integrity of SPIO-loaded RBCs. The Resovist^®^- and Sinerem^®^-loaded RBCs were slightly smaller on average than the untreated cells (MCV 79-60 versus 87 fl) with less haemoglobin per cell (MCH 20-16 versus 28 pg), but with a near normal mean cellular haemoglobin concentration (MCHC 31-28 versus 33 g dl^-1^). The Endorem^®^-loaded RBCs had an even smaller MCV (62-42 versus 87 fl), due to the higher dilution applied to RBCs during the loading procedure (given the lower Fe concentration present in the bulk suspension compared to Resovist^®^ and Sinerem^®^ suspensions), with a final MCH of 19.4-10.5 versus 28 pg and MCHC of 31.5-25 versus 33g dl^-1^.

**Figure 2:**
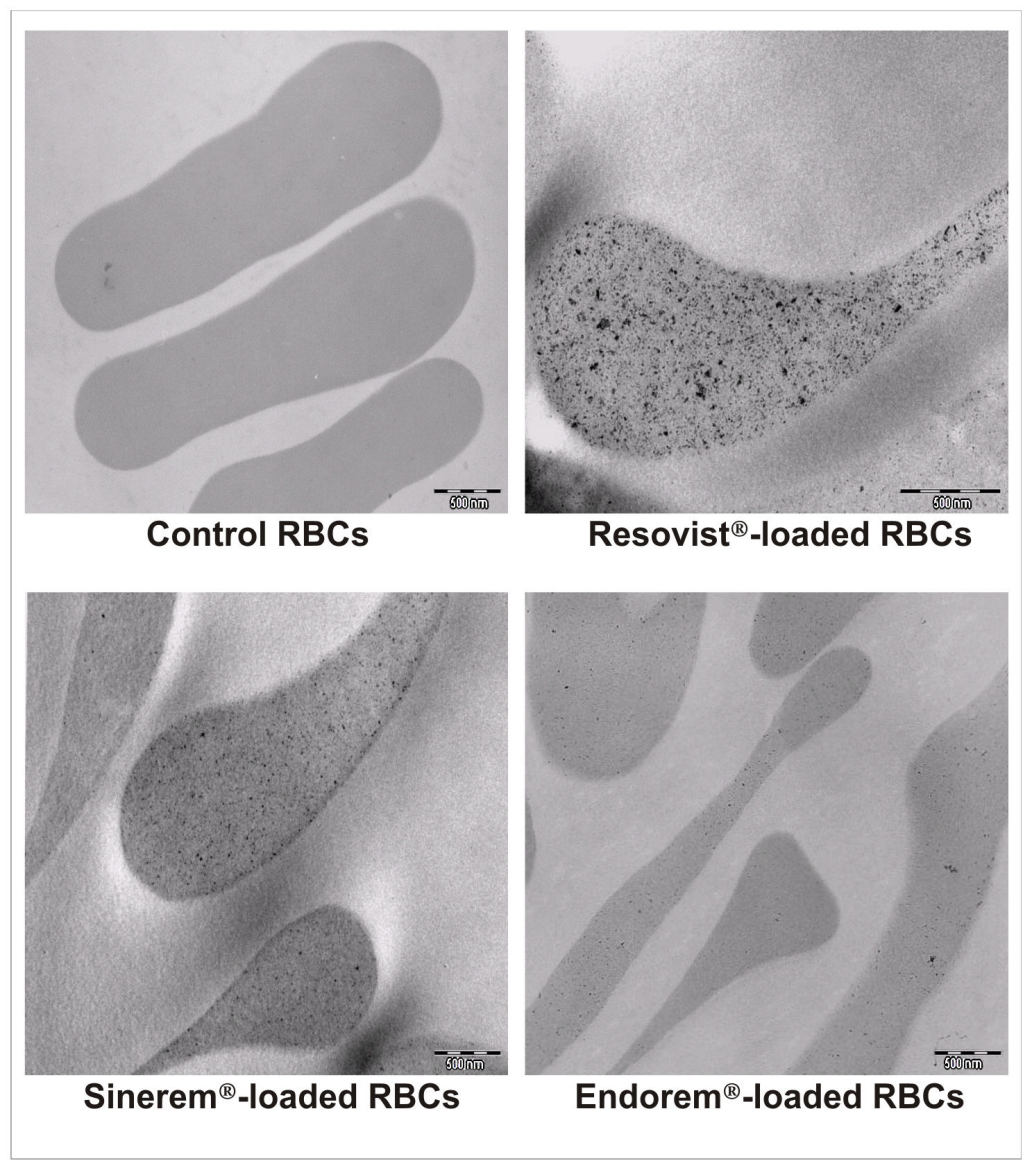
Transmission Electron Microscopy (TEM) analysis of human SPIO-loaded RBCs. TEM images show the presence of magnetic nanoparticles in RBCs loaded with Resovist^®^, Sinerem^®^ or Endorem^®^ contrast agents compared to unloaded control cells.

Electronic transmission microscope (TEM) analyses of RBCs showed the presence of magnetic nanomaterial homogeneously distributed in the cytoplasm and not bound to the membrane surface of cells (Figure 2). 

T1 and T2 relaxation time values of human Resovist^®^-, Endorem^®^- or Sinerem^®^-loaded RBCs samples resulted significantly lower than the values of control cells. It was not possible to determine the exact T2 values (<5 ms) for loaded RBC samples since they were under the instrumental sensitivity. T1 value of Resovist^®^- Endorem-^®^ and Sinerem^®^-loaded RBCs ranged from 31 to 90 ms versus 2235 ms for control cells. NMR analysis is not a direct method of iron content encapsulated in the RBCs, therefore it provide iron concentrations values that are only relative and not absolute. However, on the basis of a previous report [18], the values of iron content obtained by NMR relaxation measurements are similar to iron values measured by methods such as Vibrating Sample Magnetometer (VSM) and Inductively Coupled Plasma-Optical emission spectrometry (ICP-OES). The range of the final concentrations of magnetic material in human RBCs is 5.6-16.7 mM Fe for Resovist^®^-loaded RBCs, 7.7-11.7 mM Fe for Sinerem^®^-loaded RBCs and 5.3-8 mM Fe for Endorem^®^-loaded RBCs.

The loading procedure performed on murine blood resulted in Resovist^®^-, Endorem-^®^ and Sinerem^®^-loaded RBCs with a final cell recovery value ranging from 15 to 35%, which is much lower than the value obtained with human RBCs. This discrepancy stems from the higher osmotic fragility of murine RBCs during the hypotonic dialysis process.

In addition, the loaded murine RBCs were slightly smaller on average than the untreated cells (MCV 40 versus 50 fl), with less haemoglobin per cell (MCH 14.6 versus 16.3 pg), but with a near normal mean cellular haemoglobin concentration (MCHC 31 versus 33.3 g dl^-1^). 

TEM analyses showed intracellular presence of iron oxide nanoparticles confirming the efficiency of the encapsulation procedure of Resovist^®^, Endorem^®^, and Sinerem^®^ in murine RBCs. These findings were similar to those for loaded human RBCs (data not shown).

T1 value of Resovist^®^- Endorem-^®^ and Sinerem^®^-loaded RBCs ranged from 134 to 302 ms versus 2106 ms for control cells. The encapsulation of all these SPIO contrast agents in murine RBCs resulted in the range of 1.4-3.55 mM Fe, calculated by T1 NMR measurements.

Resovist^®^ and Sinerem^®^ contrast agents were chosen among all superparamagnetic nanoparticles tested *in vitro* to perform *in vivo* experiments.

### 3.2. Pharmacokinetic of murine Resovist^®^-loaded RBCs in mice

The murine Resovist^®^-loaded RBC sample obtained as described in “Materials and Methods” was analysed by NMR. T1 value of Resovist^®^-loaded RBCs was lower than the control value (162.16 ms versus 2106 ms) and the resulting Fe concentration encapsulated in RBCs was 3 mM Fe.

Mice receiving 1.5 Fe μmoles by intravenously injected Resovist^®^-loaded RBCs were monitored throughout the experiment; blood samples taken at different times were analysed through T1 NMR measurements in order to evaluate the survival of these SPIO-RBC carriers in the bloodstream.

**Figure 3:**
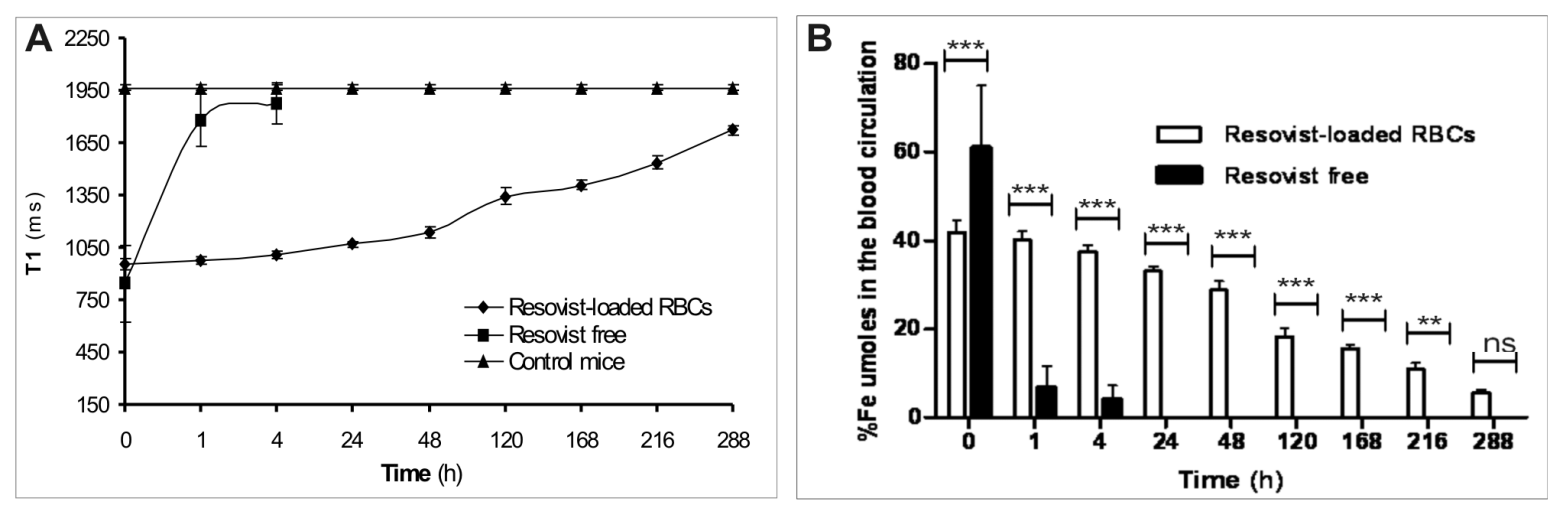
In vivo pharmacokinetic NMR results after intravenous injection in mice of murine Resovist^®^-loaded RBCs. (**A**) T1 values of blood samples from treated and control mice. (**B**) The percentage of Fe μmoles in the blood circulatory system compared to the Fe dose injected or by Resovist^®^-loaded RBCs or as free Resovist^®^. Values are expressed as means of three experiments and the error bars show the standard deviations. Asterisks indicate statistical significance; (ns) not significant; (**) p<0.01 and (***) p<0.001.

As shown in Figure 3A, blood T1 values of Resovist^®^-loaded RBC treated mice were significantly lower (from 953.6 ± 33.6 ms to 1535 ± 40 ms) than those of control mice (1965.6 ± 20.8 ms) and this lower value persists for up to 12 days, when the T1 values (1721 ± 28.6 ms) drop to values similar to those of control mice. After Resovist^®^-loaded RBC injection, the Fe μmoles present in the animal bloodstream were 0.6 at 1 hour from treatment, corresponding to 40 ± 1.9 % of the injected dose, and 0.5 after 24 hours, corresponding to approx 33 ± 1.12 % of the injected dose. 

The Fe μmoles are still detectable in the blood stream at subsequent times with percentages decreasing slowly to 5.6 ± 0.7 % for the last experimental point (Figure 3B).

On the contrary, an equal amount of free Resovist^®^ contrast agent injected in mice induces a decrease in blood T1 values, measurable at 1 hour (1777 ms ± 150) when the percentage of Fe μmoles corresponds to 6.7%, but this effect completely disappears after 4 hours (1875 ms± 117) from injection (see Figure 3, A and B).

### 3.3. Human Sinerem^®^-loaded RBCs: in vivo studies in mice

In order to increase the amount of contrast agent in the animal bloodstream we injected human SPIO-loaded RBCs rather than murine SPIO-loaded RBCs. The loading procedure with human red blood cells results in the encapsulation of higher nanoparticle concentrations thanks to their lower osmotic fragility and higher mean corpuscular volume. 

To avoid the removal by RES of injected human SPIO-loaded RBCs in mice, and consequently to improve their in vivo life span, the animals were pretreated with clodronate, a bisphosphonate that it is effective in macrophage depletion. 

The survival of human RBCs in mouse blood was first measured by cytofluorimetric analyses, after intravascular injection of human unloaded RBCs in clodronate pretreated mice (Group III) or animals not treated with clodronate (Group II). The results showed that, immediately after the injection, the percentage of human RBCs in the bloodstream of Group III mice (51.5 ± 8%) was higher than that which was found in Group II mice (27.6 ± 7.5%).

At 24h from RBCs injections, T1 and T2 NMR measurements were performed on blood samples collected from Group IV mice receiving human Sinerem^®^-loaded RBCs and clodronate pretreated Group V mice receiving the same amount of human Sinerem^®^-loaded RBCs.

As expected, the T1 and T2 values of Group II and III were not significantly different from the values of control mice (Group I) (see Table 2). 

**Table 2:** T1 and T2 NMR measurements in blood samples from ICR (**CD-1** ^®^)** mice after human RBC injections.**

**Mouse Groups**	**T1** (ms)	**T2** (ms)	[mM] from r1	**Fe μmoles in blood**
I	1990.3 ± 40.2	101 ± 7.5	/	/
II	1939.3 ± 27.7	107.5 ± 3.2	/	/
III	1966 ± 23.5	105.6 ± 1.8	/	/
IV	1033.3 ± 29	41 ± 4.2	0.216 ± 0.015	0.55 ± 0.03
V	521.2 ± 20.2	16.5 ± 2.9	0.688 ± 0.02	1.65 ± 0.05

At 24h from RBC injections, T1 and T2 relaxation times of blood samples collected from control mice (Group I), mice receiving human unloaded RBCs (Group II), clodronate treated mice receiving human unloaded RBCs (Group III), mice receiving human Sinerem^®^-loaded RBCs (Group IV) and clodronate treated mice receiving human Sinerem^®^-loaded RBCs (Group V) were measured. Values are expressed as means ± SD of three similar experiments.

On the contrary, Group IV and V mice showed T1 and T2 values significantly different from all other mice groups (p<0.001), Table 2. Moreover, T1 values of Group V mice decreased significantly (p<0.001) compared to values of Group IV mice. At 24h from Sinerem^®^-loaded RBC injection, the Fe μmoles in the blood of Group V mice, calculated from T1 NMR measurements, were 3 times higher than those found in Group IV mice, Table 2. At 24h from i.v. injection of bulk Sinerem^®^ (not encapsulated in RBCs) in mice treated or not treated with clodronate no significant differences were found between the two groups of mice (data not shown). 

**Figure 4:**
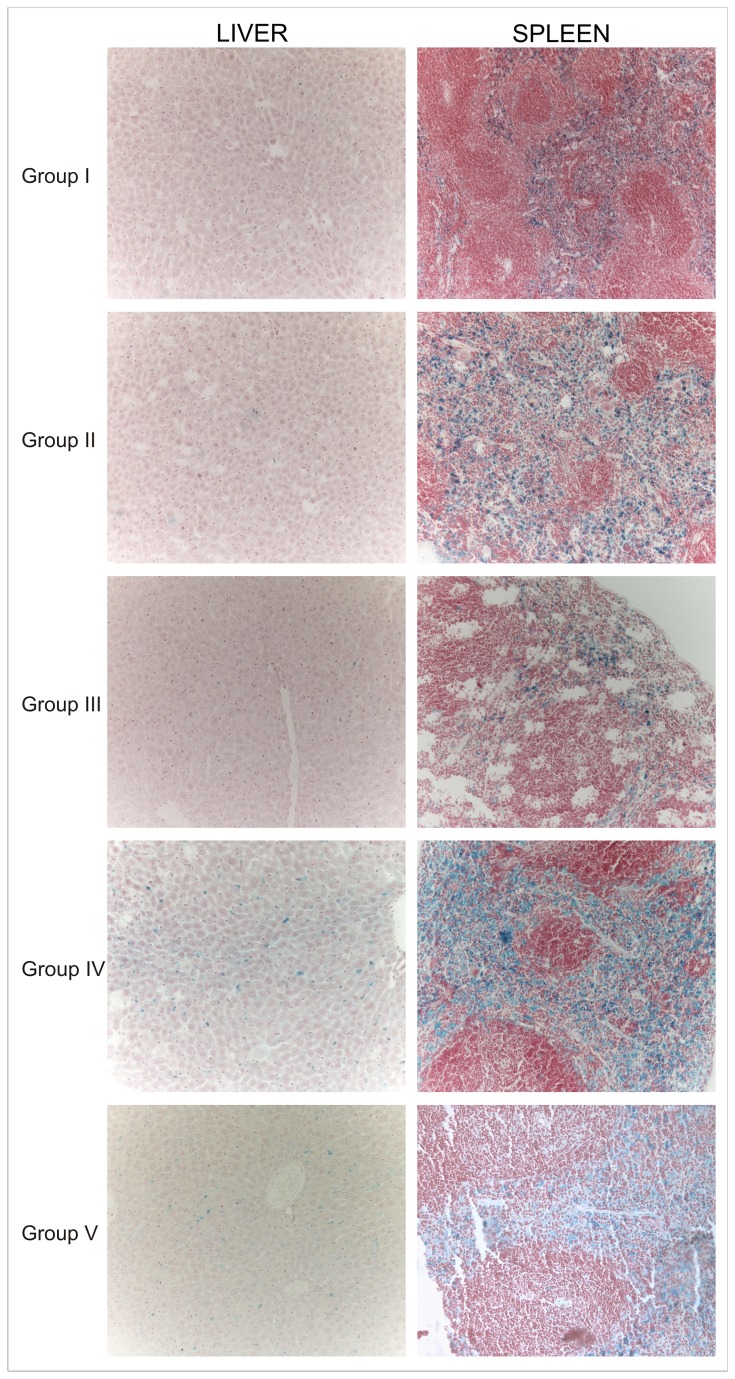
Histological sections of ICR (CD-1^®^) mice liver and spleen tissues stained by Perl’s method. Iron presence in liver and spleen sections was detected after the i.v. injection of human unloaded- or SPIO-loaded RBCs in mice treated or not treated with clodronate. *Group* *I*: control mice. *Group* *II*: mice receiving unloaded RBCs. *Group* *III*: mice pretreated with clodronate receiving unloaded RBCs. *Group* *IV*: mice receiving Sinerem^®^-loaded RBCs. *Group* *V*: mice pretreated with clodronate and receiving Sinerem^®^-loaded RBCs. Magnification 20X.

Histological examinations with haematoxylin-eosin of organs from the mice groups euthanized at 24h after unloaded and Sinerem^®^-loaded RBCs injections showed no evidence of tissue damage. Representative slices of livers and spleens stained using Perl’s method are shown in Figure 4. The liver and spleen of Group II mice displayed an iron intensity similar to Group I mice, while Group IV mice present a darker blue staining. On the contrary, Group III mice show a reduction in iron staining intensity compared to Group I and Group II mice. A strong reduction in tissue iron is evident in the liver and spleen tissue of Group V mice (Figure 4). Perl’s staining of kidneys showed a similar presence of iron in mice treated or not treated with clodronate and receiving unloaded or Sinerem^®^-loaded RBCs (Group II, III, IV, V), but the blue staining in kidney tissue was more intense than that found in control mice (Group I), (data not shown).

**Figure 5:**
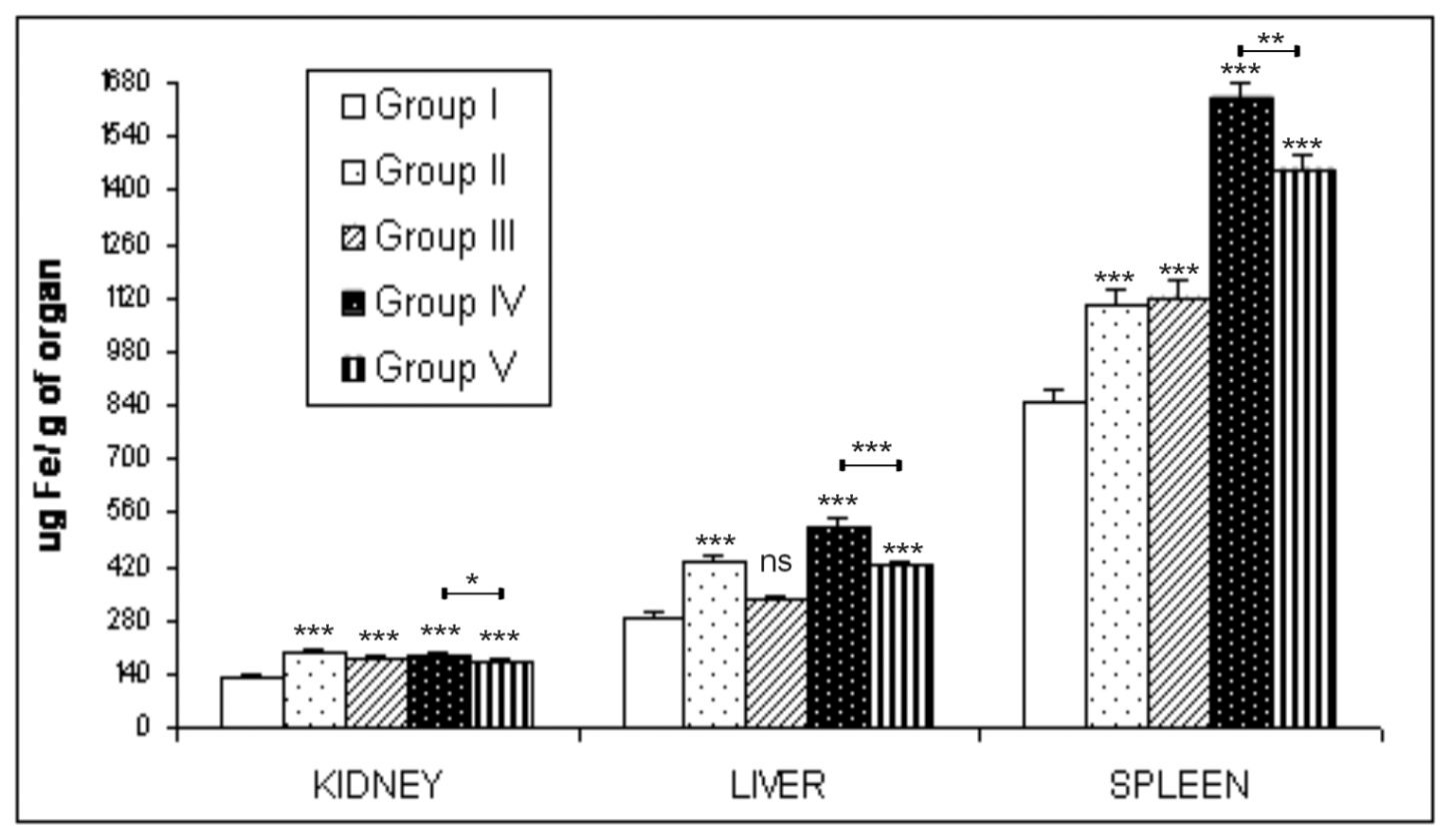
AAS analyses of Fe content in kidney, liver and spleen tissues of ICR (**CD-1^®^**) **mice**. The iron content in the organs of mice treated or not treated with clodronate before human unloaded- or Sinerem^®^-loaded RBCs intravenously administered was analysed by atomic absorption spectrometry. *Group* *I*: control mice. *Group* *II*: mice receiving unloaded RBCs. *Group* *III*: mice pretreated with clodronate receiving unloaded RBCs. *Group* *IV*: mice receiving Sinerem^®^-loaded RBCs. *Group* *V*: mice pretreated with clodronate receiving Sinerem^®^-loaded RBCs. Values are expressed as means of three experiments and the error bars show the standard deviations. Asterisks indicate statistical significance versus control group (*Group I*); (ns) not significant; (*) p ≤ 0.05, (**) p<0.01 and (***) p<0.001.

Iron concentrations in the liver, spleen and kidney tissues of mice euthanized 24h after intravenous administration of unloaded or loaded RBCs, were also determined by the flame atomic absorption spectrometry (AAS) method. Figure 5 shows iron concentrations expressed in μg/g fresh tissue and corresponding to the mean of three measurements for each animal group. 

As expected, spleen tissue of clodronate pretreated mice and animals that were not treated with clodronate receiving human RBC injections (Group II, III, IV, V) have an iron content that is significantly higher than the organs of control mice (Group I) (p<0.001). Moreover, Group II and Group III mice show a similar iron content, which is about 1.3 fold higher than that found in the spleen tissue of Group I mice. There was a marked increase in iron concentration in the spleens of Group IV and V mice receiving the injection of Sinerem^®^-loaded RBCs. Indeed, the iron content of Group IV and Group V mice is 2 and 1.5 fold higher respectively than Group I mice. Pretreatment of mice with clodronate led to a significant decrease in iron content in the spleen tissue of Group V compared to Group IV mice (p<0.01).

In liver tissue, the injection of human unloaded or Sinerem^®^-loaded RBCs in mice (Groups II, III, IV, V) led to an increase in iron content compared to control mice (Group I), Figure 5.

Moreover, livers of Group II and Group IV mice showed a significantly higher iron content than Group III and Group V mice that were clodronate pretreated before the injection of unloaded and Sinerem^®^-loaded RBCs, respectively (p<0.001). Particularly, the iron content of livers of Group IV mice is approximately 1.3 fold higher than that of Group V mice. A significant increase in the iron concentration in the kidneys of human unloaded or Sinerem^®^-loaded RBC treated mice (Groups II, III, IV, V) compared to control mice is also evident (p<0.001). The clodronate pretreatment of Group III and Group V mice resulted in a significant decrease in the iron concentration in kidney tissue compared to Group II and IV mice (p<0.05).

**Figure 6:**
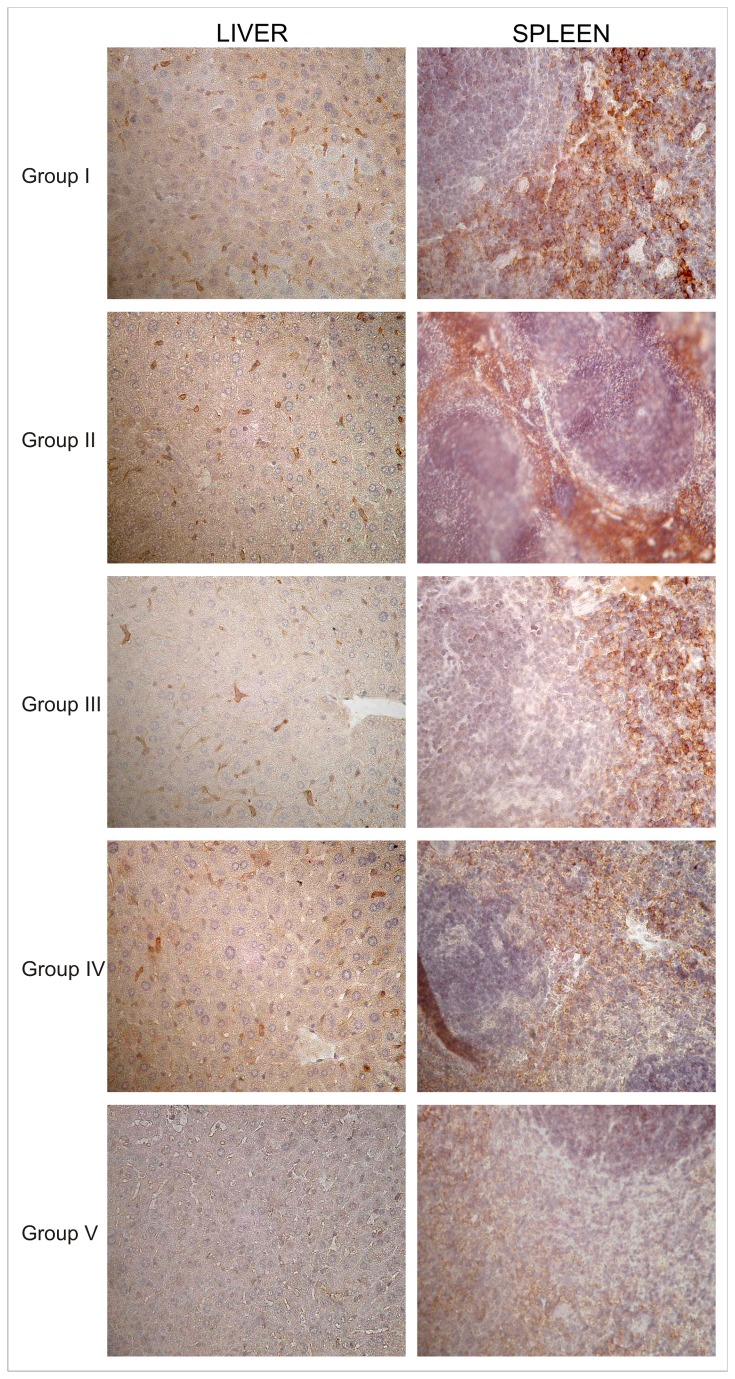
Histological sections of ICR (CD-1^®^) mice liver and spleen tissues marked with F4/80 antibody. Livers and spleens were removed 24 hours post human RBC injection and the F4/80 monoclonal antibody was used as a marker for mouse macrophages. Brown patches represent stained macrophages. *Group* *I*: control mice. *Group* *II*: mice receiving unloaded RBCs. *Group* *III*: mice pretreated with clodronate receiving unloaded RBCs. *Group* *IV*: mice receiving Sinerem^®^-loaded RBCs. *Group* *V*: mice pretreated with clodronate receiving Sinerem^®^-loaded RBCs. Magnification 40X.

Liver and spleen macrophages of mice pretreated with clodronate were evaluated by immunohistochemical localization of the macrophage-specific antigen F4/80 using a monoclonal antibody and the staining of tissue sections are shown in Figure 6. For the negative control without the primary antibody, no staining was observed (data not shown).

**Figure 7:**
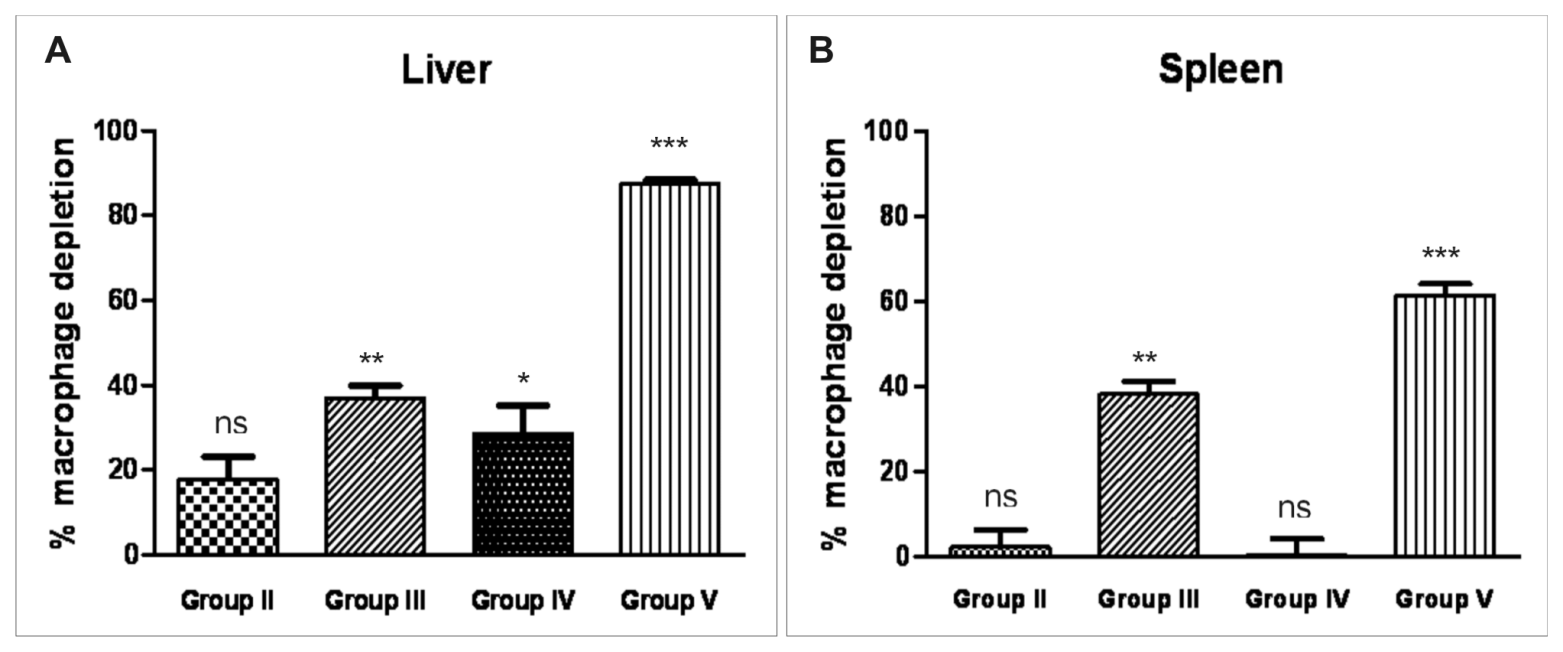
Macrophage depletion in liver and spleen tissues of ICR (CD-1^®^) mice. The number of liver (**A**) and spleen (**B**) macrophages was evaluated by counting ten fields/slides 24 hours after human unloaded- or SPIO-loaded RBC administration. The results are expressed in percentage compared to untreated control mice. Values are expressed as means of three experiments and the error bars show the standard deviations. Asterisks indicate statistical significance versus control group (*Group I*); (ns) not significant; (*) p ≤ 0.05, (**) p<0.01 and (***) p<0.001.

Mice receiving human unloaded RBCs (Group II) or human Sinerem^®^-loaded RBCs (Group IV) showed F4/80-positive spleen and liver cells, similarly to control mice (Group I). On the contrary, a marked reduction in the number of macrophages after the pretreatment with clodronate was found in Group III and V mice (Figure 6). The results showed a marked reduction in liver macrophages corresponding to 37 ± 4.95 % for Group III mice and 89 ± 3.79 %, for Group V mice (Figure 7A). The effect of clodronate pretreatment is also evident in the spleen where macrophage depletion was 38.16 ± 5.1% for Group III mice and 61.32 ± 4.74% for Group V mice (Figure 7B).

### 3.4. Human Sinerem^®^-loaded RBCs: in vivo studies in rats

The NMR measurements of blood samples drawn at 24 h from human Sinerem^®^-loaded RBC injection in clodronate pretreated rats (Group IV) and rats that were not treated with clodronate (Group III) showed T1 values lower than T1 values of untreated rats (Group I) and rats receiving unloaded RBCs (Group II). Moreover, T1 values of Group IV rats were lower than T1 values of Group III rats (data not shown). At 24h, 2.2 ± 0.2 Fe μmoles, calculated from T1 values, were present in the bloodstream of Group IV rats, 1.7 fold higher than those present in the bloodstream of Group III rats (1.3 ± 0.4 Fe μmoles). The Fe μmoles present in the blood of Group IV rats remained significantly higher for up to 5 days, while at 7 days they totally disappeared. This was also the case for Fe μmoles in the blood of Group III rats (Table 3).

**Table 3:** Fe µmoles in the bloodstream of Sprague Dawley^®^ rats treated or not treated with clodronate.

**Fe μmoles in blood**				
**Rat groups**	**24h**	**48h**	**5 days**	**7days**
III	1.3 ± 0.4	0.5 ± 0.04	0.37± 0.02	/
IV	2.2 ± 0.2	1.2 ± 0.3	0.62 ± 0.03	/

After Sinerem^®^-loaded RBC administration, blood samples were drawn at different times and Fe µmoles were calculated from T1 NMR measurements.

(Group III): rats receiving Sinerem^®^-loaded RBCs. (Group IV): clodronate treated rats receiving Sinerem^®^-loaded RBCs. Values are expressed as means ± SD of three similar experiments.

**Figure 8:**
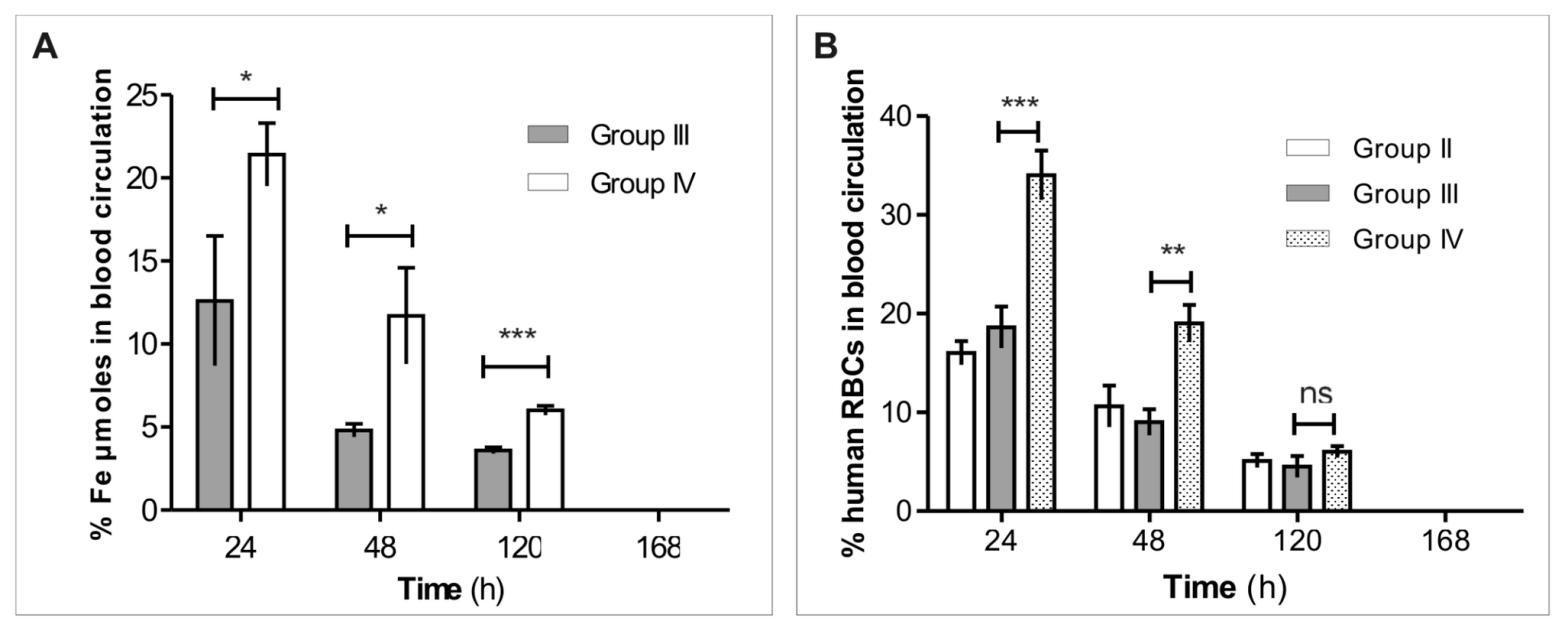
Pharmacokinetics of human Sinerem^®^-loaded RBCs in Sprague Dawley^®^ rats. (**A**) Percentage of Fe µmoles in the bloodstream of rats calculated in comparison with the injected dose after T1 NMR measurements. (**B**) Cytometric determinations of human RBC percentages present in the rat bloodstream after human unloaded- and Sinerem^®^-loaded RBC intravenous injection. *Group* *II*: rats receiving human unloaded RBCs. *Group* *III*: rats receiving human Sinerem^®^-loaded RBCs. *Group* *IV*: clodronate pretreated rats receiving Sinerem^®^-loaded RBCs. Values are expressed as means of three experiments and the error bars show the standard deviations. Asterisks indicate statistical significance; (ns) not significant; (*) p≤ 0.05; (**) p<0.01 and (***) p<0.001.

Figure 8A shows the Fe μmole percentages in the blood of Group III and IV rats calculated in comparison with the injected dose of human Sinerem^®^-loaded RBCs. After 24 hours, Fe μmole percentage in the blood of Group IV rats was about 21 % compared to 12.6 % in Group III rats.

**Figure 9:**
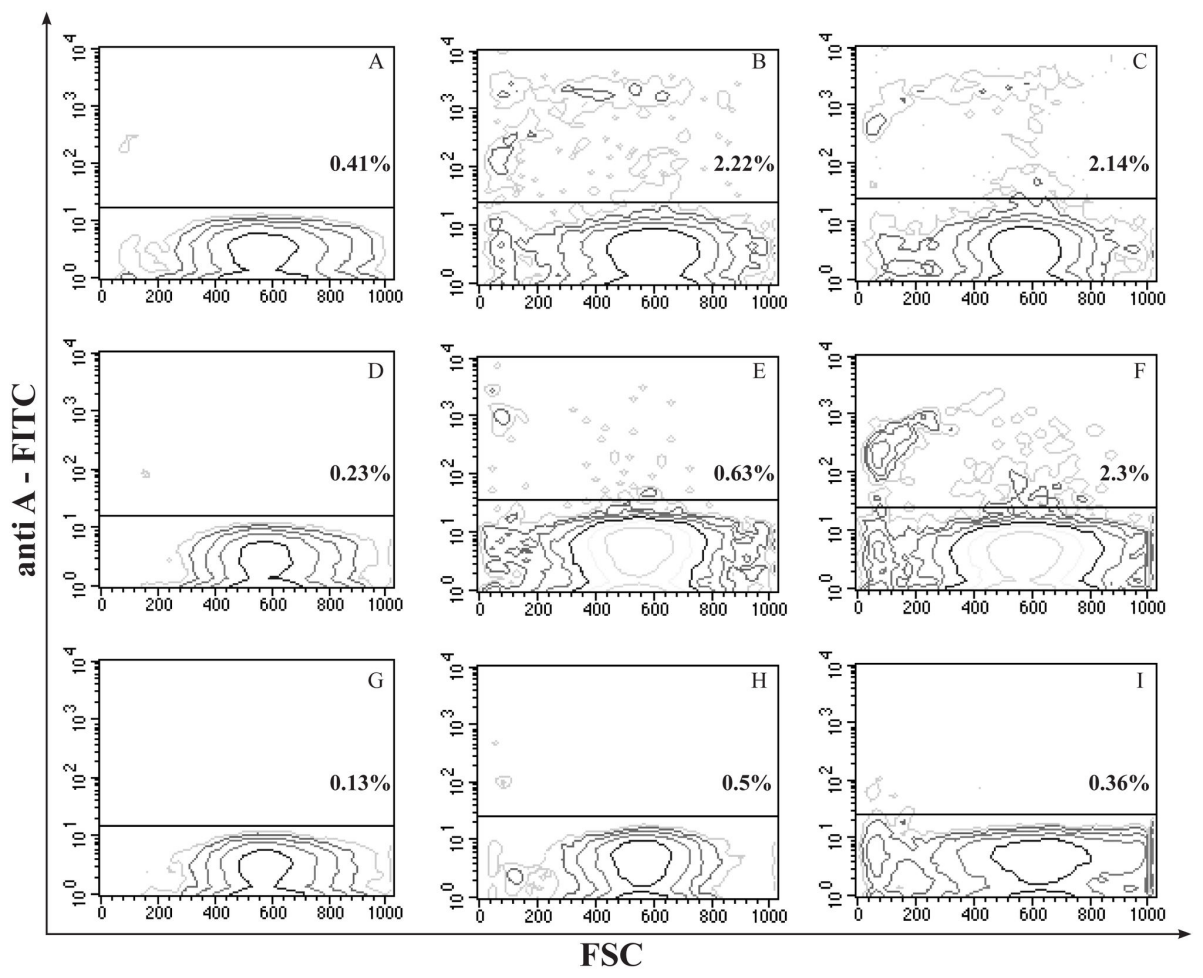
Cytometric detection of human SPIO-loaded RBCs in rat blood circulation. Contour plot FSC vs Anti-A FITC fluorescence of red blood cells from rats receiving: unloaded RBCs (Group II, left column), Sinerem^®^-loaded RBCs (Group III, middle column), and Sinerem^®^-loaded RBCs after clodronate pretreatment (Group IV, right column). Images are related to cytometry analyses of blood samples taken at 24h (A, B, C), 48h (D, E, F) and 5 days (G, H, I). The position of bars was established taking into account cellular self-fluorescence on fluorescence profile (FL1). Percentages of human RBCs positive for anti-A labelling are specified in the figures.

Moreover, the presence of human RBCs in the rat bloodstream was also evaluated by cytofluorimetric analyses. A representative cytometric analysis of blood samples drawn from Group II, Group III and Group IV rats, at 24h, 48h and 5 days in order to evaluate the presence of human RBCs is reported in Figure 9.

Based on these cytometric determinations, the percentages of human RBCs positive for anti-A labelling contained in the analyzed sample were calculated. It can be observed that at 48 h the percentage of Anti-A-positive cells is 2.3 in clodronate pretreated rats (Group IV), which is higher than the percentage (0.63) found in rats not treated with clodronate (Group III). Moreover, at 48h the percentage of human RBCs in Group III blood samples (0.63) decreased 3.5 fold compared to the percentage at 24h. On the contrary, this does not occur in Group IV, which at 48h maintains a percentage similar to that detectable at 24h (2.14). Hence, the percentages of Anti-A- positive cells were used to calculate the total number of human RBCs present in the bloodstream of the differently treated rat groups (Figure 8B) considering weight, and the number of RBCs in the total animal blood volume.

The results showed that 24h from injection, 16 ± 1.2 % of human unloaded RBCs were present in the blood circulation of Group II rats; similarly 18.6 ± 2.1 % of Sinerem^®^-loaded RBCs were detected in Group III rats. On the contrary, Group IV rats present 34 ± 2.5 % of human Sinerem^®^-loaded RBCs in their vascular system, which is twice the percentage of Sinerem^®^-loaded RBCs (18.6 ± 2.1%) present in the blood of Group III rats. The percentage values of human RBCs in blood decreased over time, finally disappearing at day 7 (Figure 8B).

It is worth noting that no animal mortality or signs of toxicity were observed during the in vivo studies involving murine and human RBCs in mice and rats. No variation in body weight or food consumption were observed during the treatment. 

## Discussion

Exogenous contrast agents are often used to enhance the diagnostic sensitivity and specificity of MRI. SPIOs are increasingly attractive for the development of MRI contrast agents since they have a higher intravascular half-life and are more sensitive than the most frequently used contrast agent gadolinium (Gd^3+^) [31].

SPIOs provide significant advantages over traditional contrast agents, including high magnetic signal strength, longer lasting contrast enhancement and relatively low cytotoxicity since the iron released from degraded SPIOs is metabolized by the body [8,13].

To be effective MR angiography contrast agents, SPIOs must be able to evade the body’s immune system so as to minimize their premature clearance from the bloodstream. Upon intravenous injection, the surfaces of nanoparticles undergo adsorption of plasma proteins, thereby triggering the opsonisation process that renders the particles recognizable by RES. Several characteristics of SPIOs, including their shape, hydrodynamic volume, surface charge and colloidal stability, have been optimized in order to minimize their clearance from the bloodstream [32,33].

Despite these efforts, SPIO contrast agents still have a short blood half-life, due to the rapid uptake by RES, which limits the applicability of such compounds for certain applications such as long term MRI monitoring. In this context, SPIO-loaded RBCs have been investigated as potential blood pool material, with longer blood retention time, for MRI/MPI diagnostic applications [18-20].

In our investigation, NMR measurements showed that SPIO-loaded RBCs, such as Resovist^®^- Endorem^®^- and Sinerem^®^-loaded RBCs, contain final Fe concentrations ranging from 5.3 to 16.7 mM for human RBCs and from 1.4 to 3.55 mM for murine RBCs. 

TEM analyses confirmed the efficiency of the procedure to encapsulate Resovist^®^, Endorem^®^ and Sinerem^®^ in human and murine RBCs showing a uniform distribution of iron oxide nanoparticles throughout the cell cytoplasm without any accumulation in the RBC membrane. This is very important because cell surface modifications could lead to a rapid elimination of RBCs by the immune system. Hence, our loading procedure yields results that are different from those described by other authors, such as Brähler et al. [34] who have encapsulated citrate-coated SPIO nanoparticles in RBCs; however, in their RBCs the iron oxide nanoparticles are also strongly attached to the external membrane. This is a restriction for the application of these loaded cells in vivo because their survival in bloodstream could be compromised by RES uptake. 

Moreover, our method produces human SPIO-loaded RBCs with a final cell recovery of around 70% without changing the main features of native cells. This percentage of cell recovery is higher than the value obtained by other authors [35,36].

We showed that iron oxide-based nanoparticles encapsulated in murine RBCs and intravenously injected in the mouse bloodstream remain in circulation longer than the free nanomaterial. Indeed, the murine Resovist^®^-loaded RBC blood retention time is longer (^~^12 days) than the free Resovist^®^ contrast agent (^~^1 h) injected at the same Fe concentration in mice (Figure 3). These results show higher *in vivo* biocompatibility and stability of the SPIO-loaded RBC constructs which preserve the main properties of the native cells able to escape the RES clearance, as well as the properties of the nanoparticles required to ensure optimal MRI performance. The potential of these biomimetic RBC constructs as new contrast agents in MRI application is already showed and MRI images of human SPIO- and USPIO-loaded RBCs were previously reported [17,37]. 

However, since the iron concentrations encapsulable in murine RBCs are lower than those attained with human RBCs, we attempted to increase SPIO nanoparticle concentration in the animal bloodstream by using of human SPIO-loaded RBCs, as larger nanoparticle carriers, intravenously injected in animals pretreated with clodronate. Moreover, the cell recovery percentage of human RBCs at the end of SPIO-loading procedure is 60-70% compared to 15-35% for murine RBCs. In fact, it is assumed that during the loading procedure with murine RBCs, a substantial number of murine SPIO-loaded RBCs will be partially damaged or lysed, due to their higher membrane fragility compared to human RBCs [38]. Therefore, we developed a new animal model which involves both the treatment of mice or rats with bisphosphonate clodronate, which is effective in temporarily suppressing macrophages, and successive intravenous injection of human SPIO-loaded RBCs containing higher nanomaterial concentrations. The intravenous administration of human red blood cells to different species such as mice and rats does not compromise the implementation of our experimental approach for *in vivo* studies; indeed, human RBCs injected into animal models do not show incompatibility with animal blood [39,40].

The results showed that the administration in mice or rats of human SPIO-loaded RBCs, such as Sinerem^®^-loaded RBCs, cause a strong decrease in animal blood T1 values.

A significant decrease in blood T1 value (521.2 ms ± 20.2), corresponding to 9.4 Fe µmoles, was observed in clodronate pretreated mice (Group V) 24 hours after human Sinerem^®^-loaded RBC injection. This value represent approximately half of the blood T1 values (1033.3 ms ± 29) found in mice receiving human Sinerem^®^-loaded RBCs but not treated with clodronate (Group IV). The corresponding Fe µmoles present in the blood of Group V mice were three times higher (1.65) than those (0.55) found in the bloodstream of Group IV mice (Table 1). Despite the injection of human Sinerem^®^-loaded RBCs (9.4 Fe µmoles), the Fe µmoles in the bloodstream of Group IV mice were present in similar amounts (0.5) to those observed 24 hours from the injection of 1.5 Fe µmoles by murine Resovist^®^-loaded RBCs (Figure 3). On the contrary, a higher value of Fe µmoles (1.65) in the bloodstream was attained when mice were pretreated with clodronate before human Sinerem^®^-loaded RBC injection. In fact, a marked reduction in the number of macrophages in liver and spleen tissue was found in mice 48 hours after the last clodronate treatment. It is known that free clodronate accumulates extensively in bone by binding to apatite crystals, but the drug also accumulates in the spleen and, to a lesser extent, in the liver of mice and rats leading to the elimination of phagocytic cells [41]. An increase in apoptotic cells in liver sections of mice treated with clodronate has been described in literature [22], and in a previous study, we investigated selective targeting of clodronate to the macrophage compartment showing a marked reduction of spleen macrophages in mice receiving free clodronate or clodronate-loaded RBCs. Moreover, no macrophages were found in liver tissue [42].

As reported in literature, macrophage subpopulations repopulate the spleens and the livers of mice or rats within 1-4 weeks after treatment with clodronate [43-45].

The data obtained by spleen and liver macrophage counts have also shown an increase in macrophage depletion after human unloaded and SPIO-loaded RBCs phagocytosis. 

RES macrophages are involved in the recognition and destruction of blood-borne pathogens but are also responsible for the clearance of damaged cells or non-self cells. In this respect, RES macrophages continuously remove senescent RBCs from the peripheral circulation but a robust erythrophagocytosis can interfere with macrophage functions. The effect of RBC phagocytosis on macrophage viability has been investigated by other authors [46] who describe how a reduction in macrophage viability and an increase in apoptosis are induced when large amounts of RBCs are ingested. Hence, the administration of human RBCs in mice appears to induce a reduction of macrophages in liver and spleen tissues. Indeed, we found a macrophage depletion of 18% in liver tissue in Group II mice receiving unloaded RBCs alone and 37% in Group III mice receiving human unloaded RBCs and pretreated with clodronate (Figure 7A). The percentage of macrophage depletion is more evident when SPIO-loaded RBCs are administered: 28.6% in Group IV mice receiving loaded RBCs and 89% in Group V mice receiving human loaded RBCs and pretreated with clodronate. A similar effect was also observed in spleen tissue. 

The marked increase in macrophage depletion in the tissues of Group V mice may be due to the high amount of iron transported by SPIO-loaded RBCs and transferred into macrophages affecting the survival of these cells. This may also explain macrophage depletion in spleen tissue. 

It has been reported in literature that the phagocytosis of extravasated red blood cells and thereby of hemoglobin, heme, and iron released after cell lysis can negatively influence the viability of cells [47]. The successful macrophage depletion, due to the pretreatment of mice with clodronate, was also confirmed by the significant decrease in the iron content of liver and spleen tissue of Group V mice compared to the iron content of Group IV mice (Figure 5). Moreover, data obtained by Perl’s staining of liver and spleen tissue of Group IV mice, showing a more intense blue staining than that found in tissues of Group V mice (Figure 4), are in agreement with AAS analyses (Figure 5).

It is also evident that iron content in liver and spleen tissue is significantly higher in Group V than in clodronate pretreated mice receiving human unloaded RBCs (Group III). Group IV also showed a significantly higher iron content in liver and spleen tissue than Group II (Figure 5). These differences can be accounted for by the injection of human Sinerem^®^-loaded RBCs which obviously contain more iron than unloaded RBCs. Moreover, iron content present in the liver tissue of Group II is significantly higher than that of Group III. On the contrary, there does not appear to be a significant difference in the iron content in spleen tissue between Group II and Group III (Figure 5).

Pharmacokinetic experiments performed on rat animal models confirm the potential of this strategy to maintain high concentrations of iron oxide-based contrast agents in blood circulation. 

The Fe µmoles in the bloodstream of rats receiving human Sinerem^®^-loaded RBCs and pretreated with clodronate (Group IV) were significantly higher than Fe µmoles present in the bloodstream of Group III rats for the first five days (Figure 8A). In fact, there were almost two fold more Fe µmoles in the blood of Group IV rats than in Group III rats (Table 3). On the other hand, the circulating human Sinerem^®^-loaded RBCs are clearly detectable by flow cytometry for up to five days (Figure 8B). 24 hours after human Sinerem^®^-loaded RBC injection, 34 % of the total injected RBCs in the blood of Group IV rats is present, compared to only 18.6% in Group III rats. These percentages decrease to 19% and 9%, respectively, after 48 hours from RBC injections and disappear completely by day 7 when human RBCs become undetectable by flow cytometry (Figure 8B). Hence, these results confirm the efficacy of clodronate treatment to preserve human loaded RBCs in the animal blood circulation.

In conclusion, we have reconfirmed the potential of our method that makes it possible to prolong the life span of iron oxide-based contrast agents through the use of red blood cells as biomimetic carriers able to survive as native functional cells. In addition, we have shown how higher Fe concentrations in animal bloodstream can be obtained using larger SPIO nanoparticle containers, such as human red blood cells, combined with a transient depletion of tissue macrophages induced by clodronate. Hence, with this approach we have further improved the results reported in our previous publications [15,20] since increased Fe concentrations in bloodstream were obtained. This animal model paves the way for the study of the use of human SPIO-loaded RBCs in MRI/MPI applications potentially allowing patients to be imaged on a number of occasions over time, especially in angiography of the circulatory system and in the detection of occluded vessels or altered angiogenesis. The use of longer lasting blood half-time contrast agents would make MRI and MPI highly suitable for perfusion imaging, image guided drug delivery or interventional applications that require repeated imaging such as those used in the diagnosis and assessment of cardiovascular diseases.
